# Breast metastasis from BRCA1-mutated ovarian cancer: response to parp inhibition and a potential lymphatic route

**DOI:** 10.1186/s12905-026-04791-7

**Published:** 2026-08-15

**Authors:** Weiwei Xie, Riqiang Liu, Zhiyong Xu, Yanyan Xie

**Affiliations:** 1 Department of Medical Oncology, People’s Hospital of Guangxi Zhuang Autonomous Region, Nanning, Guangxi 530021 China; 2 Department of Thyroid Surgery, People’s Hospital of Guangxi Zhuang Autonomous Region, Nanning, Guangxi 530021 China

**Keywords:** Ovarian cancer, Breast cancer, high-grade serous carcinoma, PAX8, GATA3, PARP inhibitor, antibody-drug conjugate, lymphatic metastasis, *BRCA1* c.964del mutation

## Abstract

**Background:**

Breast metastasis from extramammary malignancies is rare, with ovarian cancer being an exceptionally uncommon primary source. These metastases frequently mimic primary breast carcinoma, posing substantial diagnostic and therapeutic challenges.

**Case presentation:**

A 44-year-old woman presented with a breast mass initially suspected to be a primary breast carcinoma based on estrogen receptor (ER) (+, 80%), progesterone receptor (PR) (+, 5%), and Human Epidermal Growth Factor Receptor 2 (HER2) (0) staining. However, subsequent workup revealed a synchronous ovarian high-grade serous carcinoma (PAX8+/WT1+), raising the question of dual primary versus metastatic disease and prompting re-review of the breast biopsy, which ultimately confirmed the metastatic ovarian origin of the breast lesion. A pathogenic germline BRCA1 mutation c.964del (p.Ala322Leufs*19) was identified. After neoadjuvant chemotherapy and cytoreductive surgery, the patient received olaparib maintenance therapy, achieving 27 months of progression-free survival before platinum-resistant recurrence. Repeat HER2 testing of the breast lesion at recurrence showed conversion to IHC 2 + with FISH amplification, prompting enrollment in a clinical trial evaluating an anti-HER2 antibody–drug conjugate (ADC).

**Conclusions:**

This case highlights three key points. First, the PAX8/WT1/GATA3 immunohistochemical panel is critical for distinguishing ovarian metastasis from primary breast carcinoma. Second, the route of spread from the ovary to the breast, possibly transdiaphragmatic via the cardiophrenic and internal mammary nodes, requires further investigation. Third, PARP inhibitor maintenance in the context of a germline BRCA1 mutation provided sustained disease control, and HER2 status conversion at recurrence opened a therapeutic window for sequential antibody–drug conjugate (ADC) therapy.

## Background

Metastasis to the breast from extramammary malignancies is a rare clinical entity, with solid tumor metastases accounting for approximately 0.75%–1.76% of all breast malignancies [1, 2]. The reported incidence varies across studies, likely reflecting differences in case ascertainment and study populations. The most frequent primary tumor types include melanoma, lung, gastrointestinal carcinomas, and gynecologic carcinomas [1, 3, 4]; however, their relative frequencies vary by geographic region. For instance, in Southern China, nasopharyngeal carcinoma is a recognized source of metastatic disease [5]. Among gynecologic malignancies, ovarian carcinoma is an exceptionally rare source of breast metastasis, with only sporadic cases documented in the literature. Ovarian cancer remains a leading cause of gynecological cancer-related mortality [6]. Approximately 70%–80% of cases are histologically classified as high-grade serous carcinoma (HGSC), often associated with germline BRCA1/2 mutations [7–9]. Most patients present with advanced-stage disease. Standard management typically involves cytoreductive surgery and platinum-based chemotherapy, often followed by maintenance therapy with PARP inhibitors with or without bevacizumab [7]. Despite high initial response rates, most patients eventually relapse owing to the emergence of chemoresistance. The management of platinum-resistant disease remains challenging, prompting the investigation of novel strategies, including antibody-drug conjugates (ADCs) [7, 10]. Herein, we report a rare case of synchronous breast metastasis arising from a primary ovarian carcinoma. The diagnostic trajectory was complicated by an atypical presentation that closely mimicked that of primary breast cancer. A definitive diagnosis was achieved using a multidisciplinary approach integrating imaging, histopathology, and immunohistochemical profiling. Notably, longitudinal imaging provided findings suggestive of a potential metastatic pathway from the adnexal primary tumor to the breast via the cardiophrenic and internal mammary lymph nodes.

## Case presentation

This report follows the CARE (Case Report) guidelines for the presentation of case reports. A 44-year-old woman presented to our hospital in December 2022 with a 4-day history of worsening shortness of breath. Her obstetric history was G2P1A1. She had no history of oral contraceptive use or a prior diagnosis of malignancy. Her father had been diagnosed with lung cancer, and her sister was diagnosed with fallopian tube cancer approximately 26 months after the patient’s initial diagnosis (the sister did not undergo genetic testing because of financial constraints, and her BRCA status remains unknown).

Physical examination revealed a mass in the inner lower quadrant of the right breast and hard, non-tender axillary lymph nodes, with no overlying skin changes or peau d’orange. No obvious masses were observed in the left breast. Chest CT revealed a large right pleural effusion, multiple space-occupying lesions involving the right breast and axillary lymph nodes, and mediastinal lymphadenopathy. Thoracentesis yielded pleural fluid with cytology positive for adenocarcinoma cells. Ultrasound-guided core-needle biopsy of the right breast mass revealed invasive carcinoma of no special type (NST). Immunohistochemical (IHC) analysis showed pan-cytokeratin (+), calponin (−), CK5/6 (−), ER (+, 80%), PR (+, 5%), HER2 (0), Ki-67 (80%), and E-cadherin (+), initially suggesting primary breast carcinoma.

**Fig. 1:**
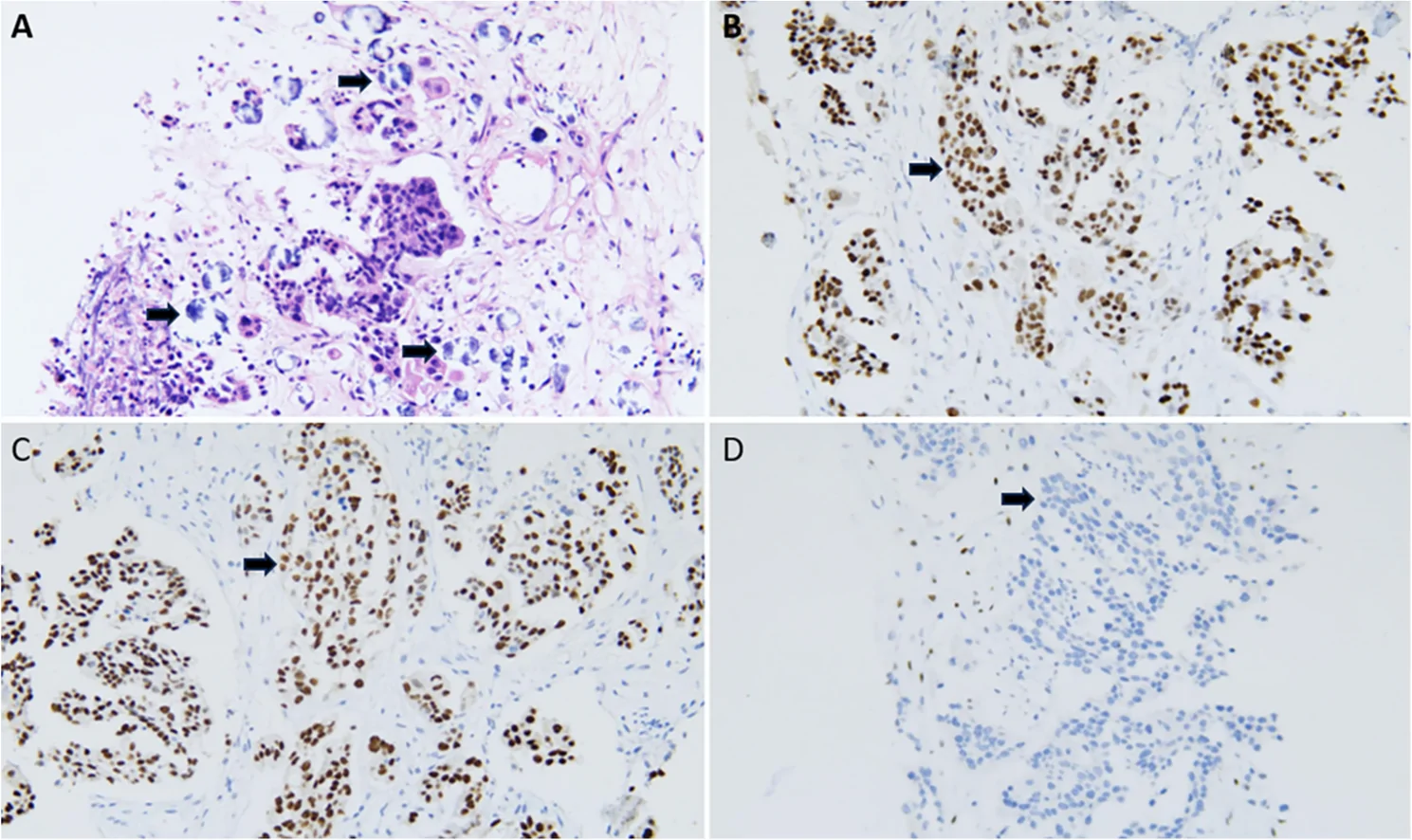
Metastatic carcinoma with papillary architecture and psammoma body (arrow) (**A**) (100×). Immunohistochemistry shows strong nuclear positivity for PAX8 (**B**) and WT1 (**C**), with negative staining for GATA3 (D) (200×)

Staging abdominopelvic CT revealed a large right pelvic mass with multiple metastases involving the omentum, pelvic peritoneum, diaphragmatic surface, liver capsule, and enlarged regional lymph nodes. Ultrasound-guided biopsy of the pelvic mass revealed high-grade serous carcinoma (HGSC) with a predominant micropapillary architecture on hematoxylin and eosin staining. IHC analysis revealed that the tumor cells were positive for PAX8, WT1, CK7, and ER (+, 20%). p53 showed a mutant pattern with diffuse and strong nuclear positivity in > 95% of tumor cells. Ki-67 was 60%, and HER2 was 1+. The staining results were negative for PR, GCDFP-15, GATA-3, SATB2, HNF-1β, and CD20. This immunoprofile supported a Müllerian origin and was consistent with the diagnosis of HGSC. Subsequent immunostaining of the breast biopsy revealed PAX8 (+), WT1 (+), and GATA-3 (−), confirming that the breast and axillary lesions were metastases from the primary ovarian HGSC rather than a synchronous primary breast cancer (immunohistochemical comparisons are shown in Fig. 1).

The final diagnosis was FIGO stage IVB HGSC of the right ovary, characterized by a rare germline BRCA1 c.964del (p.Ala322Leufs*19) variant, a concomitant germline RECQL4 c.1652 C > T (p.Ala551Val) variant, and a homologous recombination deficiency (HRD)-positive phenotype. Genetic testing was performed by Surexam Medical Laboratory (Guangzhou, China) using a hybridization capture-based next-generation sequencing (NGS) hereditary cancer gene panel. The BRCA1 c.964del (p.Ala322Leufs*19) variant was detected at a variant allele frequency (VAF) of 45.92% with a sequencing depth of 956×, consistent with a heterozygous germline mutation.

### Treatment and follow-up

**Fig. 2:**
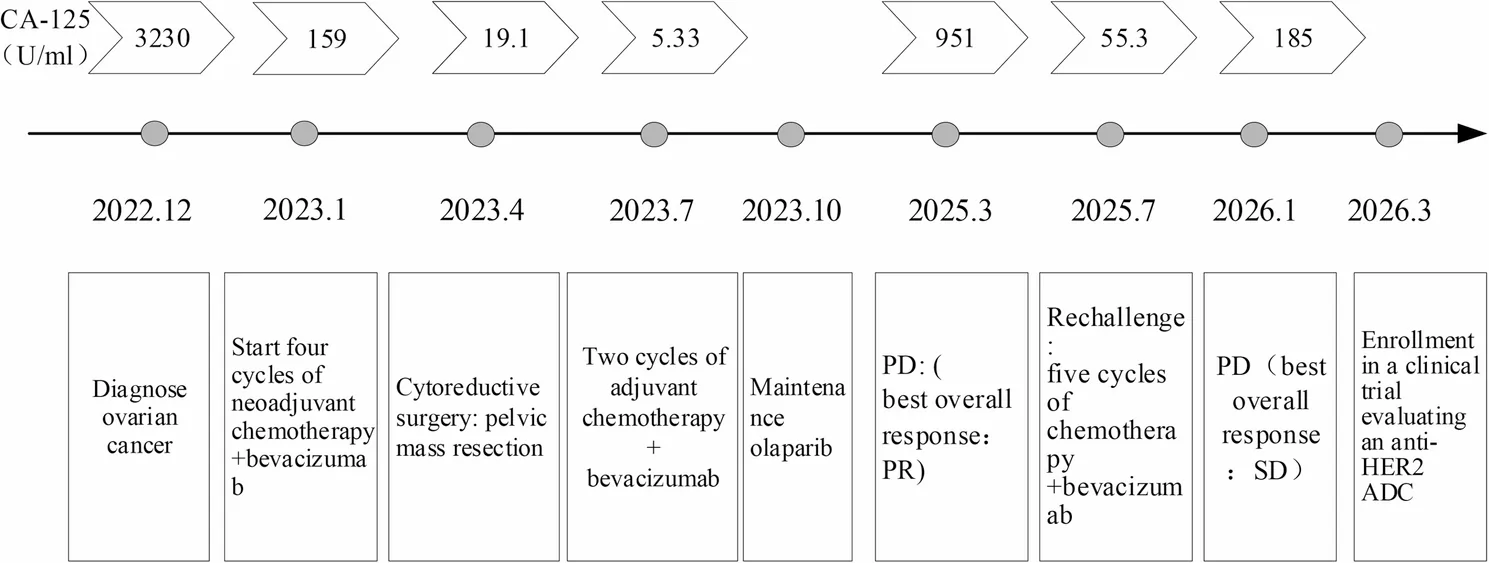
Clinical timeline. Key events and corresponding CA-125 levels (U/mL) are shown chronologically. PR, partial response; SD, stable disease; PD, progressive disease

The patient received neoadjuvant chemotherapy with paclitaxel liposome (175 mg/m²), carboplatin (AUC 5), and bevacizumab (15 mg/kg) administered every 21 days, achieving a partial response after four cycles. She underwent interval cytoreductive surgery with no gross residual disease, followed by two additional cycles of adjuvant chemotherapy. Maintenance olaparib (300 mg twice daily) was initiated as monotherapy; concomitant bevacizumab maintenance was not pursued because of financial constraints. Grade 3 anemia and fatigue necessitated dose reduction to 150 mg twice daily after four months, which was subsequently well tolerated. The patient responded to treatment, with a decline in CA-125 levels, and remained progression-free for 27 months. In March 2025, she experienced disease progression with enlargement of the breast mass. Rechallenge with the prior platinum-based regimen resulted in stable disease as the best response, with disease progression after five cycles. Repeat biopsy of the breast mass showed HER2 IHC 2 + with FISH positivity (HER2/CEP17 ratio 3.05, average HER2 copy number 10.7 signals/cell, positive per the 2023 ASCO/CAP criteria). These findings prompted enrollment in a clinical trial evaluating an anti-HER2 ADC (NCT number: 06834672). Figure 2 shows the Clinical timeline of diagnosis, treatment, and disease course.

## Discussion

Metastasis to the breast from extramammary malignancies is rare, with solid tumor metastases accounting for only 0.75% to 1.76% of all breast malignancies, and ovarian primaries specifically representing approximately 0.07% of breast malignancies [11]. Epithelial ovarian cancer (EOC), particularly high-grade serous carcinoma (HGSC), poses a significant diagnostic challenge when involving the breast owing to substantial histopathological and immunohistochemical overlap with primary breast adenocarcinoma [12–15]. It has been demonstrated that providing pathologists with a patient’s oncologic history improves diagnostic accuracy from 36% to 98% (*p* < 0.0001) [12]. Anatomically, ovarian carcinoma typically disseminates via transperitoneal, hematogenous, and lymphatic routes [16, 17]. The rarity of mammary involvement may stem from the relatively low vascularity of the breast. When metastasis does occur, it primarily proceeds through hematogenous or lymphatic routes [17, 18]. Hematogenous seeding usually manifests as well-circumscribed solitary masses, often in the upper outer quadrant, whereas lymphatic involvement tends to present as diffuse parenchymal infiltration [3, 17, 18].

### Proposed lymphatic pathway

**Fig. 3:**
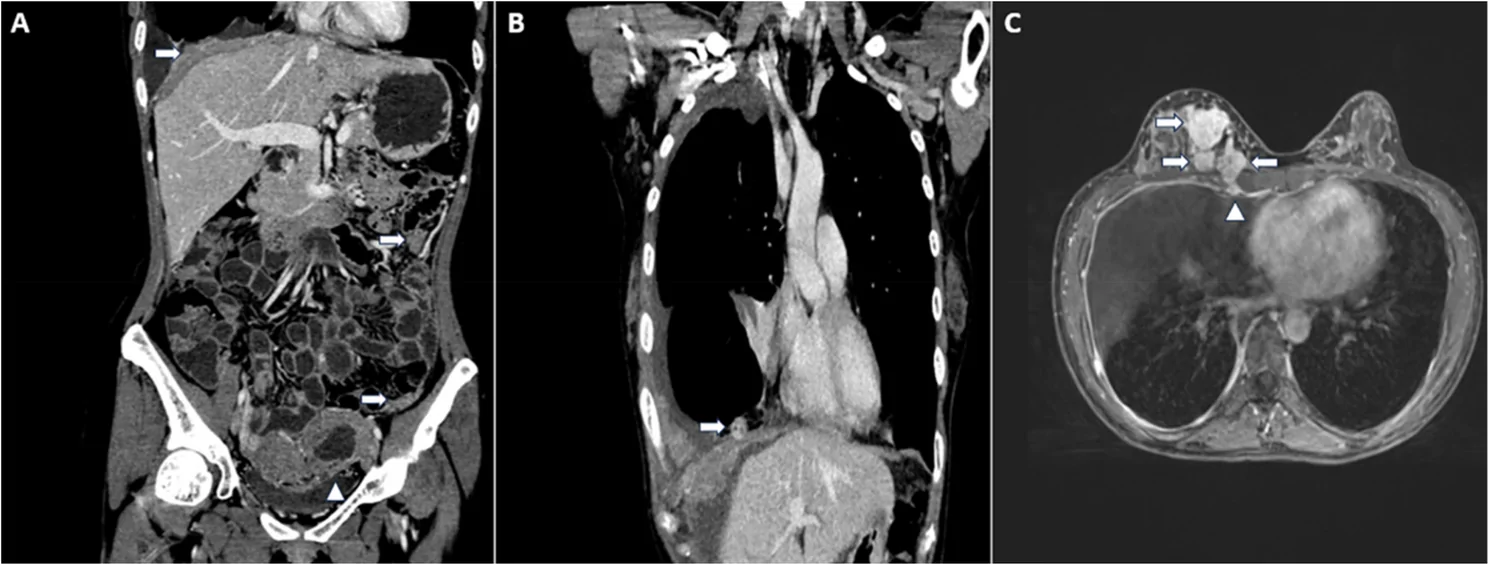
Proposed pathway of ovarian metastasis to the breast. **A** Contrast-enhanced CT shows a right ovarian mass (arrowhead) with extensive peritoneal and diaphragmatic metastases (arrows). **B** Contrast-enhanced CT shows cardiophrenic angle lymphadenopathy (arrow). **C** Contrast-enhanced MRI shows breast metastases as multiple beaded nodules (arrows) and internal mammary lymphadenopathy (arrowheads)

**Fig. 4:**
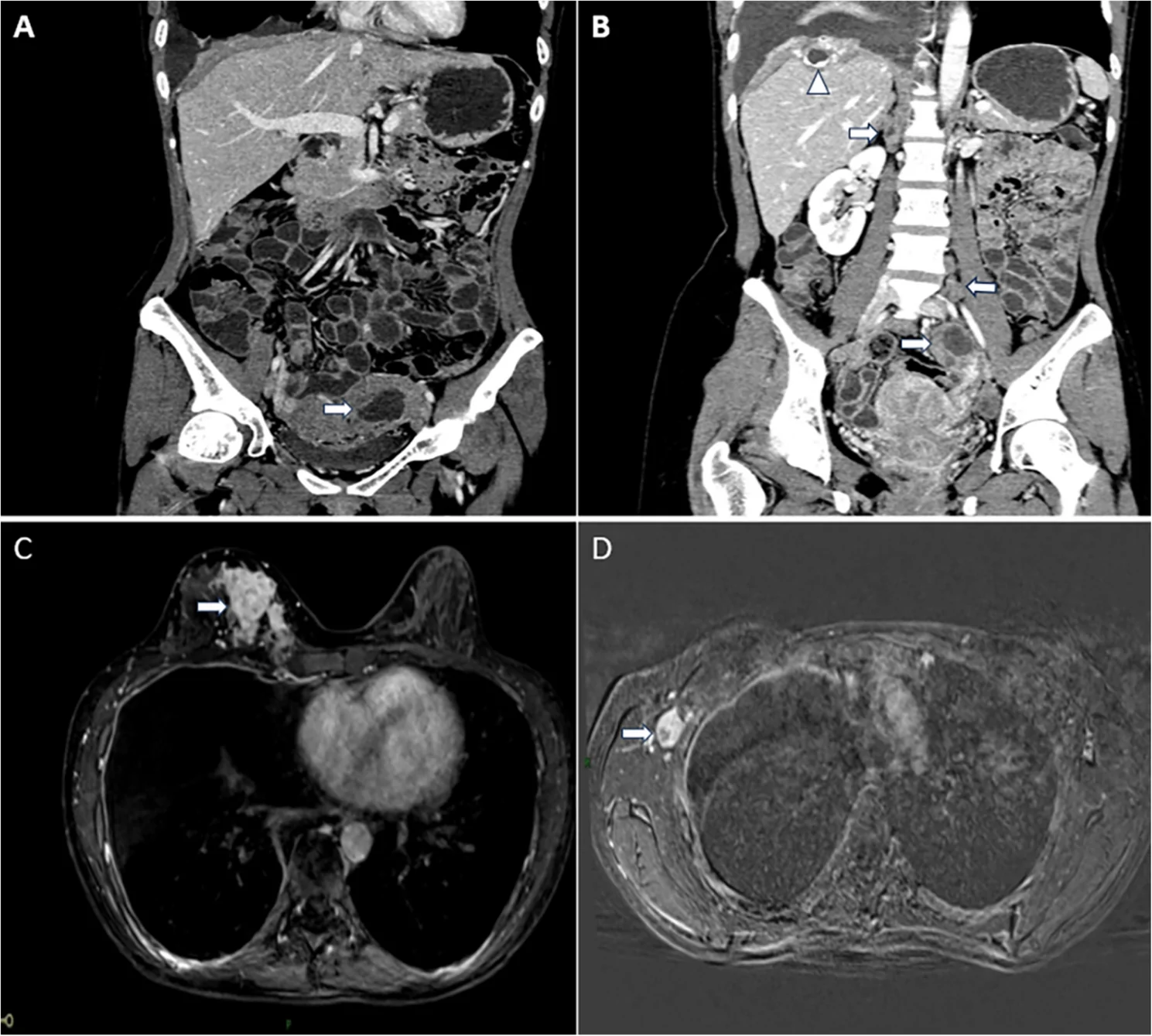
Imaging findings of primary and metastatic lesions. **A** Contrast-enhanced CT shows an ovarian mass with internal cystic degeneration (arrow). **B** Contrast-enhanced CT shows enlarged abdominal lymph nodes with cystic degeneration (arrow) and diaphragmatic metastases with calcification and cystic degeneration (arrowhead). **C** Contrast-enhanced MRI shows breast and axillary metastases with cystic degeneration (arrowheads)

**Fig. 5:**
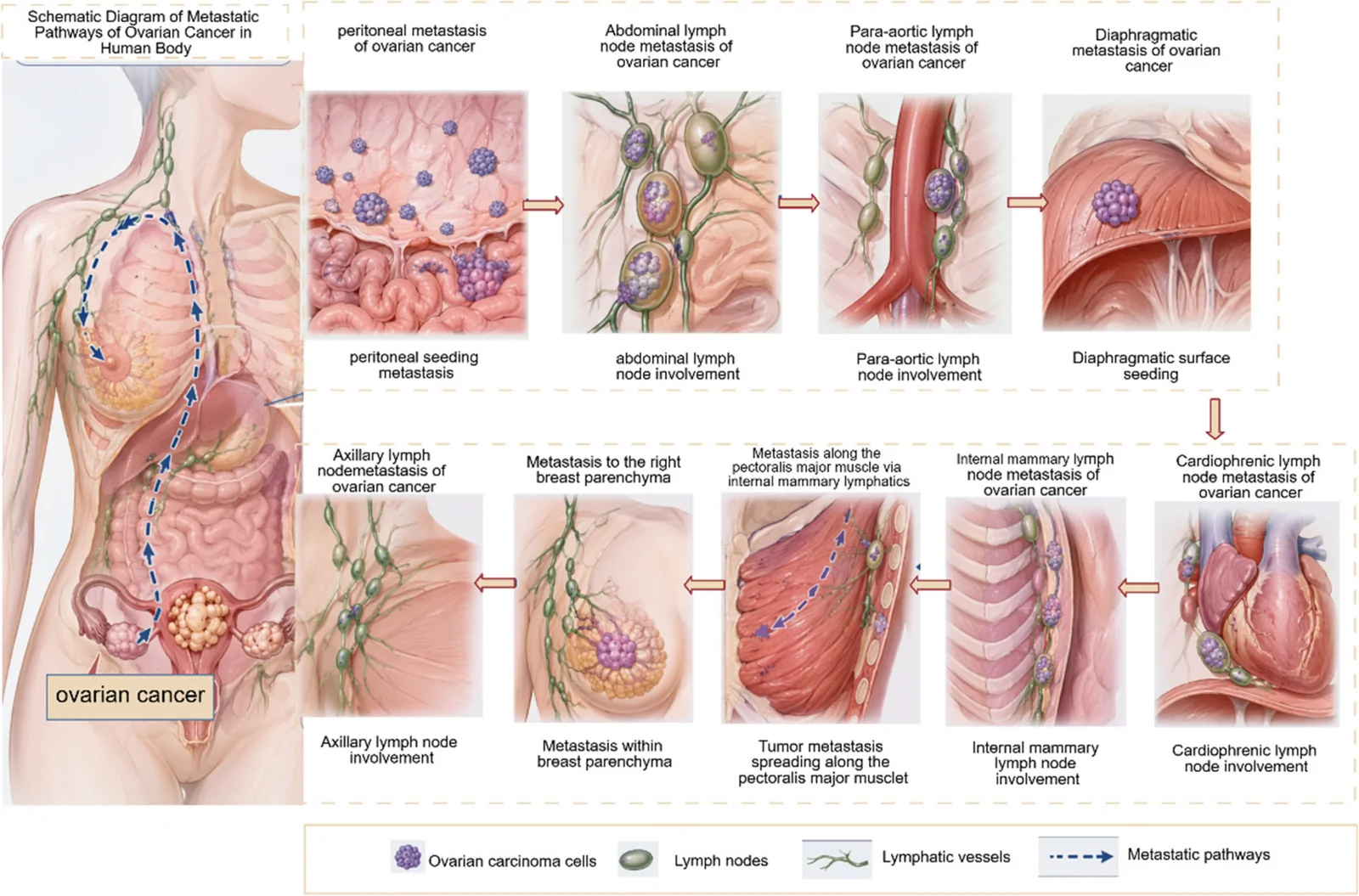
Schematic illustration of the proposed transdiaphragmatic metastatic pathway from the ovarian primary to the breast via cardiophrenic and internal mammary lymph nodes (Created with OpenAI)

In this case, the metastatic lesions were uniquely situated in the lower inner quadrant as multiple well-defined nodules arranged in a linear beaded pattern (Fig. 3C). Abdominopelvic CT revealed extensive peritoneal carcinomatosis (Fig. 3A), while chest CT identified pleural metastases and involvement of the cardiophrenic lymph nodes (CPLNs) (Fig. 3B). The CPLNs drain the diaphragm, pleura, and anterior abdominal wall, with efferent lymphatics eventually emptying into the internal mammary chain. Both CT and MRI revealed a continuous chain of internal mammary lymphadenopathy contiguous with the breast mass. While cystic changes and calcifications may occur in both primary and metastatic breast malignancies, their concordant presence in both the ovarian primary and the breast lesions provided additional radiological support for shared histogenesis, thereby reinforcing the metastatic nature of the breast findings (Fig. 4). Based on these findings, we propose one plausible anatomical explanation: peritoneal dissemination may have reached the cardiophrenic angle nodes, with possible subsequent extension to the internal mammary chain and possible retrograde dissemination to the mammary parenchyma (Figs. 3 and 5) [16]. To our knowledge, such a potential transdiaphragmatic lymphatic route has not previously been described radiologically. The linear beaded pattern of metastatic nodules and the contiguous lymphadenopathy from the cardiophrenic to internal mammary nodes would be more consistent with lymphatic spread than with hematogenous dissemination, which typically manifests as solitary, well-circumscribed masses. We acknowledge that this remains a hypothesis based on imaging contiguity rather than direct lymphatic mapping, and that lymphangiographic, pathological, or functional evidence would be required to confirm it.

### Hypothetical molecular drivers of extra-abdominal dissemination

Beyond the anatomical route delineated above, the biological triggers that facilitate such widespread extra-abdominal dissemination warrant consideration. We stress that the following discussion is presented solely as a literature-based hypothesis; androgen receptor (AR) immunohistochemistry was not performed in this patient, and no AR expression data are available. Emerging evidence suggests that loss or downregulation of AR expression may promote distant, extra-abdominal dissemination in epithelial ovarian cancer by relieving epithelial-to-mesenchymal transition suppression and accelerating tumor de-differentiation [19, 20]. Notably, AR loss and BRCA1-driven homologous recombination deficiency both converge on genomic instability and aggressive clonal evolution; whether these pathways cooperate to enable transdiaphragmatic spread remains an untested hypothesis. In the absence of patient-level AR data, this endocrine-molecular mechanism remains entirely conjectural and cannot be regarded as a confirmed driver of the unusual metastatic trajectory observed here.

### Diagnostic immunohistochemistry

The patient’s synchronous presentation without known oncologic history created a significant diagnostic challenge. The histopathological similarities initially obscured the true origin; however, the presence of scattered psammoma bodies—a hallmark of serous ovarian carcinoma—within the breast biopsy (Fig. 1A [21]), ultimately pointed toward the correct diagnosis. Initial breast biopsy showed ER (+, 80%), which can be misleading given that ER is expressed in both mammary and gynecologic malignancies. Definitive differentiation was achieved using an expanded IHC panel: tumor cells were strongly positive for PAX8 and WT-1 but negative for GATA-3 (Fig. 1). PAX8 is expressed in nearly 100% of gynecological malignancies and is virtually always negative in breast cancers [22, 23]. Although WT1 can be expressed in rare breast cancers, the PAX8+/WT1+/GATA3- profile effectively excludes a mammary primary, as GATA-3 is highly specific for breast carcinoma [24–28]. Therefore, we recommend this three-marker panel for differentiating breast metastasis of ovarian origin from primary breast cancer.

### Treatment and HER2 conversion

Historically, breast metastasis from ovarian cancer carries a dismal prognosis, with a median survival of 14–16 months [5, 13, 17]. However, approximately 50% of HGSC cases harbor homologous recombination deficiency (HRD), predominantly driven by BRCA1/2 mutations, which confers heightened sensitivity to platinum agents and PARP inhibitors [7–9]. In such patients, the 5-year overall survival rate can approach 70% [7–9]. Our patient harbored a rare germline loss-of-function BRCA1 variant c.964del (p.Ala322Leufs*19) that resulted in biallelic inactivation via loss of heterozygosity (LOH) of the wild-type allele, the canonical mechanism in hereditary BRCA1-associated carcinogenesis. No somatic BRCA1 mutation was identified in the tumor. This BRCA1-driven HRD phenotype, together with the concomitant somatic TP53 mutation, underpinned the severe genomic instability observed in this tumor and rendered it highly sensitive to platinum-based chemotherapy and PARP inhibitors. Consistent with this well-established molecular profile, our patient achieved a progression-free survival of 27 months, surpassing typical outcomes despite high-risk features, including synchronous distant metastasis and a high Ki-67 index (80%).

At recurrence, repeat HER2 testing on the breast lesion revealed conversion from 0/1 + at diagnosis to 2 + with FISH amplification. The mechanism underlying this phenotypic shift remains undetermined; this conversion may reflect the selection of pre-existing HER2-amplified subclones under therapeutic pressure, as the genomic instability inherent to HRD-positive tumors generates extensive intratumoral heterogeneity that provides a substrate for clonal evolution. This clonal evolution model is supported by recent multi-region sequencing studies in ovarian tumors, which demonstrated that histologically distinct components share truncal mutations yet harbor divergent genetic alterations, consistent with lineage-specific clonal selection [29]. Furthermore, longitudinal profiling of ADC targets in ovarian cancer has revealed that therapeutic pressure can drive directional changes in HER2 expression, providing direct evidence for antigen evolution under treatment selection [30]. Alternatively, the breast microenvironment may uniquely select for HER2-driven proliferation, given the established role of HER2 in mammary epithelial biology. Sampling variability between biopsies may also contribute. Indeed, the frequency of intratumoral HER2 heterogeneity in ovarian cancer has been systematically evaluated using dual-block assessment, with discordance rates reaching 21.4% in ovarian tumors; importantly, this approach reclassified 13.5% of cases from HER2-0 to HER2-expressing tumors [31], underscoring the practical limitation of single-biopsy assessment. This phenotypic shift prompted enrollment in a clinical trial evaluating an anti-HER2 ADC. In the DESTINY-PanTumor02 trial, trastuzumab deruxtecan demonstrated meaningful activity in HER2 IHC 2 + ovarian cancer (ORR 36.8%; mOS 13.0 months), establishing HER2-low expression as a targetable entity [32]. Beyond the pan-tumor trial data, case reports have documented durable partial responses to T-DXd in patients with HER2-low ovarian carcinosarcoma and recurrent ovarian large-cell neuroendocrine carcinoma, suggesting that the therapeutic benefit of HER2-directed ADCs extends to a broad spectrum of ovarian cancer histotypes with low-level HER2 expression [33, 34]. Our case supports the practice of repeated HER2 testing at recurrence, as it may identify actionable therapeutic targets in advanced BRCA-mutated ovarian cancer with breast metastasis.

### Familial implications and cascade screening

The identification of a rare pathogenic germline BRCA1 c.964del (p.Ala322Leufs*19) mutation in this patient has profound implications for her family members. BRCA1-associated hereditary breast and ovarian cancer syndrome follows an autosomal dominant inheritance pattern, conferring a 50% transmission risk to each first-degree relative. Notably, the patient’s sister was diagnosed with fallopian tube cancer approximately 26 months after the index case; however, genetic testing could not be completed in the sister due to financial constraints, which underscores persistent barriers to equitable access to genomic medicine and highlights a missed opportunity for risk-reducing surgery and PARP inhibitor eligibility assessment. Current guidelines from the National Comprehensive Cancer Network (NCCN) and the European Society for Medical Oncology (ESMO) recommend predictive genetic testing for all first-degree relatives of BRCA1/2 mutation carriers [9]. The concomitant germline RECQL4 c.1652 C > T variant identified in our patient has uncertain clinical significance; while biallelic RECQL4 mutations cause Rothmund-Thomson syndrome with increased cancer susceptibility, the effect of monoallelic variants on BRCA1-associated cancer risk remains uncharacterized. We emphasize that the proband’s confirmed BRCA1 c.964del variant should trigger systematic cascade testing across all at-risk relatives, thereby aligning with the paradigm of precision prevention in hereditary cancer syndromes.

## Conclusion

This case underscores the necessity of integrating clinical history, systemic imaging, and a core immunohistochemical panel (PAX8/WT1/GATA3) to avert diagnostic pitfalls. To our knowledge, this case presents imaging findings suggestive of a potential transdiaphragmatic lymphatic pathway from the peritoneum to the breast via cardiophrenic and internal mammary nodes, though this interpretation awaits confirmation by direct lymphatic mapping or histopathological studies. While breast metastases from ovarian cancer historically signify poor outcomes, the integration of multidisciplinary care and novel agents, including PARP inhibitors and ADCs, can achieve extended disease control in appropriately selected patients, particularly those with BRCA mutations.

## Data Availability

No datasets were generated or analysed during the current study.

## Abbreviations

ER: Estrogen receptor
PR: Progesterone receptor
PARP: Poly (ADP-ribose) Polymerase
ADC: Antibody-drug conjugate
HGSC: High-grade serous carcinoma
BRCA: Breast Cancer Susceptibility Gene
HRD: Homologous Recombination Deficiency
FISH: Fluorescence in situ hybridization
HER-2: Human Epidermal Growth Factor Receptor 2
EOC: Epithelial ovarian cancer
IHC: Immunohistochemistry
CPLNs: Cardiophrenic lymph nodes
PAX8: Paired box gene 8
WT1: Wilms’ tumor 1
GATA3: GATA-binding protein 3
PFS: Progression-free survival
ORR: Objective response rate
mOS: Median overall survival
